# Enoxaparin for VTE thromboprophylaxis during inpatient rehabilitation care: assessment of the standard fixed dosing regimen

**DOI:** 10.1186/s40360-023-00728-0

**Published:** 2024-01-10

**Authors:** Amir Haim, Orli Avnery, Deborah Rubin-Asher, Hagay Amir, Kaifa Hashem, Harel Ben Zvi, Motti Ratmansky

**Affiliations:** 1 Loewenstein Rehabilitation Medical Center, 278 Achuza St, Raanana, POB 3, 43100 Israel; 2 School of Medicine, Tel Aviv University, Tel Aviv, Israel; 3 Hematology Institute, Meir Medical Center, Kfar Saba, Israel

**Keywords:** Enoxaparin, Rehabilitation, Venous pulmonary embolism, Venous thromboembolism

## Abstract

**Background:**

We aimed to examine the efficiency of fixed daily dose enoxaparin (40 mg) thromboprophylaxis strategy for patients undergoing inpatient rehabilitation.

**Methods:**

This was an observational, prospective, cohort study that included 63 hospitalized patients undergoing rehabilitative treatment following sub-acute ischemic stroke (SAIS) or spinal cord injury (SCI), with an indication for thromboprophylaxis. Anti-Xa level measured three hours post-drug administration (following three consecutive days of enoxaparin treatment or more) was utilised to assess in vivo enoxaparin activity. An anti-Xa level between 0.2-0.5 U/ml was considered evidence of effective antithrombotic activity.

**Results:**

We found sub-prophylactic levels of anti-Xa (<0.2 U/ml) in 19% (12/63). Results were within the recommended prophylactic range (0.2-0.5 U/ml) in 73% (46/63) and were supra-prophylactic (>0.5 U/ml) in 7.9% (5/63) of patients. Anti-Xa levels were found to inversely correlate with patients’ weight and renal function as defined by creatinine clearance (CrCl) (*p*<0.05).

**Conclusions:**

Our study confirmed that a one-size-fits-all approach for venous thromboembolism (VTE) prophylaxis may be inadequate for rehabilitation patient populations. The efficacy of fixed-dose enoxaparin prophylaxis is limited and may be influenced by renal function and weight. This study suggests that anti-Xa studies and prophylactic enoxaparin dose adjustments should be considered in certain patients, such as those who are underweight, overweight and or have suboptimal renal function.

**Trial registration:**

No. NCT103593291, registered August 2018.

**Supplementary Information:**

The online version contains supplementary material available at 10.1186/s40360-023-00728-0.

## Key points

• Clinicians should be aware that fixed dose enoxaparin prophylaxis will only provide adequate therapeutic response for a proportion of rehabilitation patients.

• The efficacy of fixed-dose enoxaparin prophylaxis is limited and may be influenced by renal function and weight.

• A personalized approach to VTE prophylaxis that includes anit-Xa studies and prophylactic dose adjustments should be considered in certain patients, such as those who are underweight, overweight and or have suboptimal renal function.

• More studies are required to investigate the interaction of weight and creatinine in order to establish VTE prophylactic dosing guidelines for specific rehabilitation populations.

**Supplementary Information:**

The online version contains supplementary material available at 10.1186/s40360-023-00728-0.

## Introduction

Venous thromboembolism (VTE), comprising deep venous thrombosis (DVT) and venous pulmonary embolism (PE), is a common and preventable complication in patients undergoing rehabilitation, which can result in significant morbidity and death [1–7]. Prolonged bed rest (>14 days) is associated with a five-fold increased risk of developing DVT [4]. The American Society of Haematology (ASH) 2018 guidelines for the management of VTE states that the risk of VTE persists for 45-60 days following discharge from acute hospitalisation [8]. The rate of DVT has been reported as high as 33% among people undergoing rehabilitation for a variety of conditions [2]. The current study focused on two common clinical rehabilitation in-patient scenarios: patients with subacute ischemic stroke (SAIS) or recovering from acute spinal cord injury (SCI). The phase of subacute ischaemic stroke refers to the time period between one to six months of onset [8]. There is a large body of evidence that describes the prevalence of VTE in these areas [1, 3, 9–12]. Depending on the diagnostic methods and time of evaluation, the incidence of DVT without prophylaxis is 20-50% following stroke and 60-80% following SCI [1, 13]. Risk factors for developing DVT in these patients include immobility, older age (>65 years), female gender, obesity (body mass index (BMI) ≥25kg/m^2^), active cancer and muscle weakness [1, 3, 9].

The symptoms of DVT and PE are often non-specific, especially in patients undergoing rehabilitation who may under-report symptoms due to aphasia, cognitive impairment or altered conscious states [2]. VTE often presents as simply fever and leg edema [14]. Due to the difficulty associated with diagnosis, administration of routine prophylactic therapy is recommended by multiple guidelines for patients hospitalized for rehabilitation for a variety of diagnoses [10, 15, 16]. VTE prevention therapy for survivors of ischemic stroke is recommended for the duration of in-patient rehabilitation or until the stroke survivor regains mobility [3]. Based on the severity of the SCI, low molecular weight heparin (LMWH) are recommended for up to 24 weeks after injury [13].

Enoxaparin, a LMWH, is considered the standard prophylactic treatment for patients undergoing rehabilitation for SAIS or SCI [3, 10, 15]. Enoxaparin is a convenient option for thromboprophylaxis due to its predictability in regards to dose response, long half-life and lower risk of bleeding for a given antithrombotic effect [17]. Several doses and treatment regimens have been proposed; however, a fixed dose of 40mg once daily is the accepted, prevailing approach and is considered the standard prophylactic dose [12, 18, 19]. In clinical practice, a fixed dose regimen is frequently used, with the exception of patients who are underweight, obese, pregnant, or suffering from renal insufficiency (creatinine clearance (CrCl) <30 ml/min) [20, 21].

The anti-factor Xa (anti-Xa) assay is a functional assay that helps measure antithrombin (AT)-catalyzed inhibition of factor Xa by Unfractionated Heparin (UFH) and direct inhibition of factor Xa (FXa) by enoxaparin. As a result, the anti-Xa level reflects the in vivo pharmacological activity of enoxaparin. For prevention of DVT/PE, steady state peak anti-Xa activity measured three hours post-drug administration following three consecutive days of enoxaparin treatment or more is considered to be 0.2-0.5 IU/ml [22, 23].

Several studies examining anti-Xa activity with the fixed enoxaparin prophylactic dosing have reported shortcomings for several clinical scenarios. For example, critically ill, trauma and burn, plastic surgery, renal and oncology patients often demonstrate low plasma anti-factor Xa (anti-Xa) levels, suggesting inadequate prophylaxis (sub/supra prophylaxis) and which may lead to an increased risk of thrombotic and bleeding events [19, 20, 22, 24–29]. To the best of our knowledge, similar investigations have not been reported in the literature for patients in a rehabilitation setting.

The current study was devised to examine the efficiency of fixed dose enoxaparin as a thromboprophylaxis strategy for patients recovering from SAIS or SCI admitted to a rehabilitation hospital. The goal of this study was to evaluate in-vivo enoxaparin activity in these patients, and to evaluate the influence of co-variables (e.g., renal function, weight) on the outcome of this treatment strategy.

## Patients and methods

This study was an observational, prospective cohort study approved by the Institutional Ethics Committee on Human Research at the Loewenstein Rehabilitation Medical Center (No. NCT103593291). Consecutive patients hospitalized for rehabilitation following SAIS or SCI were recruited after giving consent to participate in the study.

Prophylactic treatment with subcutaneous injection of LMWH (enoxaparin 40 mg once daily) was initiated during pre-rehabilitation acute care. It was re-evaluated on admission to our rehabilitation hospital and continued where clinically necessary [3, 10]. Inclusion criteria included: age 20-80 years, hospitalization for rehabilitation following SAIS or SCI, indication for thromboprophylaxis due to major restriction of mobility and no elevated risk of bleeding. The exclusion criteria included: patients in whom there was a severe risk of bleeding, obesity (>150kg), low weight (<45kg for women, 57kg for men), renal insufficiency (CrCl <30ml/min) and patients receiving anticoagulant therapy for concomitant pathologies (e.g. atrial fibrillation). For patients recovering from SAIS, exclusion criteria also included hemorrhagic stroke, hemorrhagic transformation of stroke or bleeding into another site. All patients’ charts were reviewed for this study from the day of admission to 4-5 weeks post-admission, depending on the length of hospitalization. Any thrombotic or hemorrhagic events from admission or follow-up were collected.

### Data collection

All information was obtained from the patients’ electronic or hardcopy files. The following variables were recorded on admission and discharge: patient characteristics (age, gender, weight, height and diagnosis), days from stroke onset to admission to rehabilitation hospital and length of stay in rehabilitation hospital. A specific data collection form was developed by the research coordinator. Trained research associates, who were familiar with the process of chart review, extracted information from charts using the form provided.

Blood sample for peak plasma levels of anti-Xa activity were drawn three hours post-enoxaparin administration (after a minimum of three days of treatment). Blood samples were collected in 3.2% sodium citrate tubes and centrifuged at twice 1500g for 15min and stored at −70◦C. At this time, blood samples for serum creatinine (CR) was collected as well. Renal function was assessed by calculating CrCl according to the Cockcroft and Gault equation [30].

Plasma samples were tested for the chromogenic anti-Xa activity assay with HemosIL Liquid anti-Xa (Instrumentation Laboratory, Bedford, USA) reagent using the analyzer system ACL TOP Family 500/550 (Instrumentation Laboratory) [31]. The method is based on adding the patient’s plasma to a test tube containing FXa reagent and a chromogenic substrate to FXa. If the patient’s plasma contains enoxaparin, a complex of the drug will be formed with the patients ATIII and FXa inhibition will occur. The remaining free FXa will bind to the chromogenic substrate. The color released is measured and is inversely related to the amount of anti-Xa present in the plasma. The anti-Xa activity measurement result was a surrogate marker of enoxaparin activity (IU/ml) [31].

There is a lack of consensus regarding the recommended target levels of anti-Xa for prophylactic enoxaparin. However, generally accepted target levels between 0.2 IU/mL and 0.5 IU/mL are derived mostly from non-critically ill surgical patient population [22, 32, 33].

The target anti-Xa level was defined as 0.2–0.5 U/ml and results were divided into three categories: LOW-sub-prophylactic (<0.2 U/ml), NORMAL-prophylactic (0.2-0.5 U/ml) and HIGH-supra-prophylactic (>0.5 U/ml).

### Statistical analysis

Data were evaluated by SPSS software for Windows version 27.0 (SPSS Inc., Chicago, IL, USA). Univariate statistics included *t* test for independent groups to compare between the two studies. One-way ANOVA was used to compare between the three groups of anti-Xa (LOW, NORMAL, HIGH) for continuous variables. Pearson’s Chi-square test was used to compare categorical variables. Values were reported as mean ± SEM. Pearson’s correlation test was used to assess correlations. Significance level was set at *p*<0.05. Multiple regression was used as multivariate analysis. The dependent variable was the level of anti-Xa and the independent variables were the significant variables from the univariate analysis.

## Results

From August 2018 to November 2019, all patients hospitalized for rehabilitation following SAIS or SCI and receiving SC enoxaparin 40mg/day thromboprophylaxis were considered for inclusion. A total of 63 patients (31 SAIS and 32 SCI) were enrolled in the study (all eligible candidates who gave informed consent). Patient characteristics of the study cohorts are shown in Table 1. Most of the patients were male (70%), with a mean age of 59 years, a median creatinine clearance of 93 ml/min and a median total body weight of 75 kg. Patients in the SCI group were significantly younger than the SAIS group (mean SAIS 63.6 years, SCI 54.7 years, *p*=0.01). The percentage of patients with type 2 diabetes mellitus was significantly higher in the SAIS group (*p*=0.002). No other significant differences were found between the groups. Neither major bleeding episodes nor VTE-related events were recorded during the hospitalization period, the median follow-up time was 3.7 weeks.

**Table 1:** Characteristics of study participants

	**All (** ***n*** **=63)**	**SAIS (** ***n*** **=31)**	**SCI (** ***n*** **=32)**	**p**
Age (years) (mean)	59.1±14	63.6±7.7	54.7±17.1	0.01
Male (% of patients)	44 (70%)	23 (74%)	21 (66%)	NS
Height (m) (Median)	1.7 (0.12)	1.69 (0.12)	1.7 (0.13)	NS
Total body weight (kg) (median)	75 (21)	77 (20)	73 (14.9)	NS
BMI (kg/m^2^) (median)	26 (5.46)	27.6 (4.57)	26.2 (6.17)	NS
Diabetes (% of patients)	30 (48%)	21 (68%)	9 (28%)	0.002
Current smoker (% of patients)	20 (32%)	13 (42%)	7 (22%)	NS
Creatinine (mg/dl) (mean)	0.9±0.2	0.9±0.1	0.8±0.2	NS
CrCl (ml/min) (mean)	96.9±29.3	90.2±21.2	103.3±34.6	NS

Quantitative data are expressed as median and interquartile range or mean±standard deviation as appropriate

*BMI* Body mass index, *CrCl* Creatinine clearance computed according to the Cockroft-Gault formula

Mean peak anti-Xa levels were 0.31±0.11 IU/ml for the combined study group, 0.33±0.95 IU/ml for the SAIS cohort and 0.29±0.13 for the SCI cohort (*p*=0.199). The results showed that only 73% (46/63) of the patients studied had anti-Xa activity within the recommended prophylactic range (0.2-0.5 U/ml). In the remaining 27% of patients, enoxaparin anti-Xa activity levels were outside the recommended range, either sub-prophylactic anti-Xa activity (<0.2 U/ml) in 19% (12/63) of patients or supra-prophylactic (>0.5 U/ml) in 7.9% (5/63) (Table 2).

**Table 2:** Baseline characteristics of patients with anti-Xa levels within range compared to patients with anti-Xa levels out of the recommended range

		**Sub-prophylactic (A)**	**Within range (B)**	**Supra-prophylactic (C)**		
		<0.2 IU/mL	0.2-0.5 IU/mL	>0.5 IU/mL	p (ANOVA)	**Post Hoc Tukey's method**
n		12	46	5		
Age (years)	mean	51.4	60.8	61.2	0.1071	
	std	14.3	13.7	11.6		
BMI (kg/m^2^)	mean	28.8	27.1	20.8	0.0054	C < A,B
	std	6.1	4.0	5.2		
Creatinine (mg/dl)	mean	0.9	0.9	0.9	0.9815	
	std	0.2	0.2	0.2		
eGFR (ml/min/1.73m2)	mean	126.7	94.7	67.6	0.0009	A > B,C
	std	43.2	28.0	12.7		
Total body weight (kg)	mean	90.1	76.5	58.6	0.0001	C < B < A
	std	16.4	12.4	11.1		
CrCl (ml/min)	mean	121.3	93.7	68.2	0.0006	A > B,C
	std	27.9	26.8	13.0		
Height (m)	mean	1.8	1.7	1.7	0.0109	A > B,C
	std	0.1	0.1	0.2		
					***p (Pearson Chi-Square)***	
Male	n	11	31	2		
	Percent	25.0%	70.5%	4.5%	0.084	
Diabetes	n	4	23	3		
	Percent	13.3%	76.7%	10.0%	0.498	
Current smoker	n	2	18	0		
	Percent	10.0%	90.0%	0.0%	0.093	

**Fig. 1:**
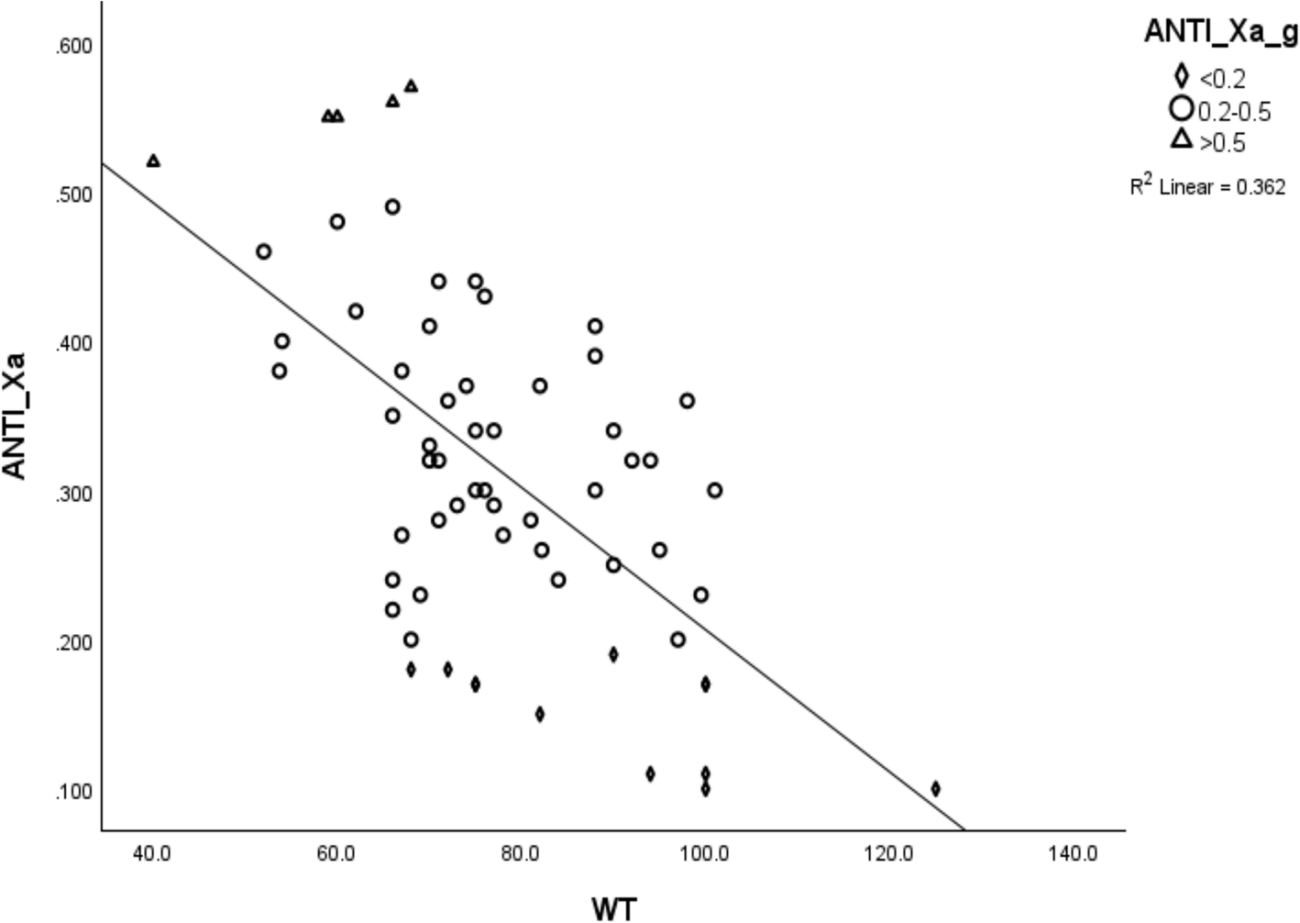
Relationship between weight (kg) and anti-Xa activity (U/ml). Scatter plot demonstrating the relationship between the categorical value of weight and anti-Xa activity. As can be seen in the graph, weight had a strong negative correlation with anti-Xa activity

**Fig. 2:**
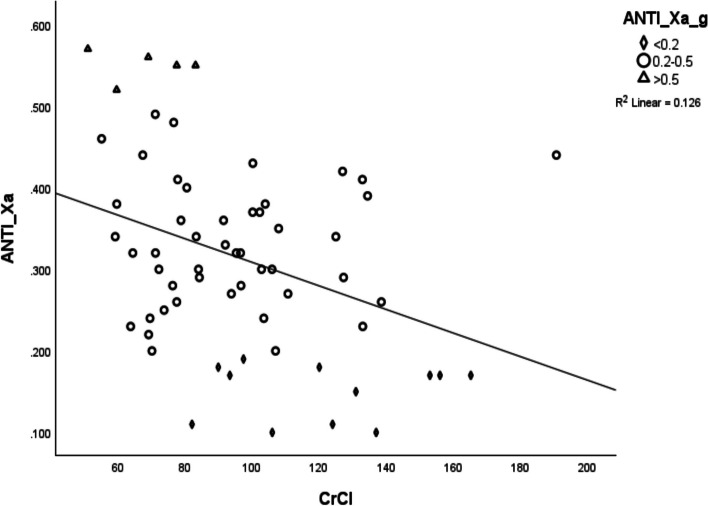
Correlation between CrCl (ml/min/1.73m^2^) and anti-Xa activity (U/ml). Scatter plots demonstrating the relationship between CrCL and anti-Xa activity. CrCl had a moderately strong negative correlation with anti-Xa activity

**Fig. 3:**
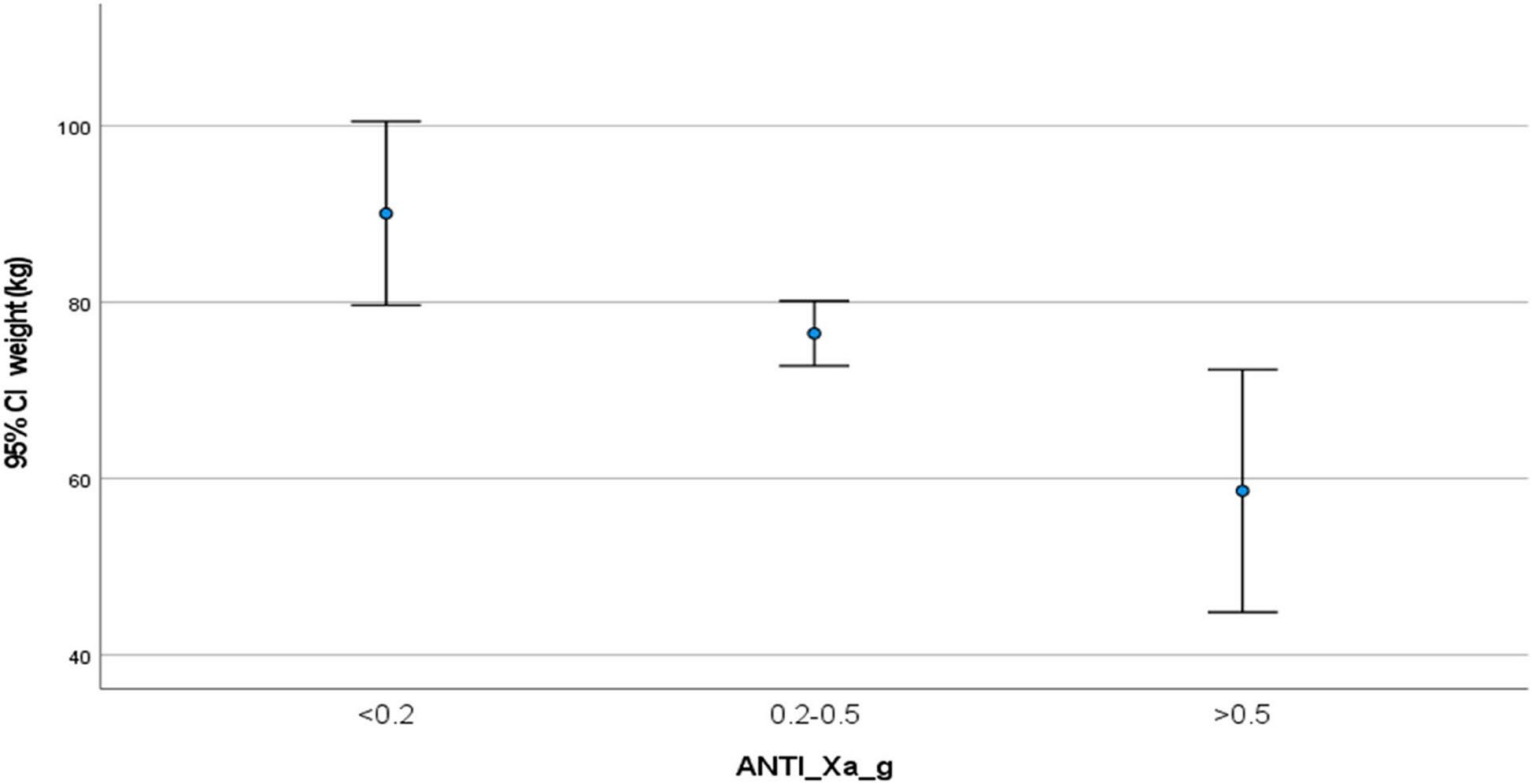
Association between weight (kg) and anti-Xa activity (U/ml). Mean values and standard deviation for weight (95% CI) compared to anti-Xa activity. Anti-Xa results were divided into three categories; subtherapeutic (<0.2 U/ml), therapeutic (0.2-0.5 U/ml) and supratherapeutic (>0.5 U/ml)

**Fig. 4:**
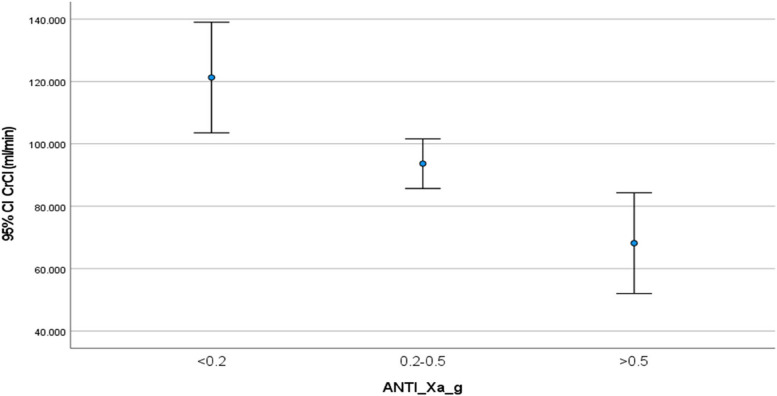
CrCl (ml/min/1.73m^2^) and anti-Xa activity (U/ml). Mean values and standard deviation for CrCl (ml/min) and anti-Xa activity (U/ml) categorized into sub-prophylactic anti-Xa activity (<0.2 U/ml), within the recommended prophylactic range (0.2-0.5 U/ml) and had supra-prophylactic (>0.5 U/ml) range

Univariate analysis of patient-related variables demonstrated a significantly negative association between weight (*r*=-0.6, *p*<0.0001), height (*r*=-0.35, p<0.005), BMI (*r*=-0.42, p<0.001) and CrCl (*r*=-0.35, *p*<0.004) with anti-Xa activity (Table 3, Figs. 1 and 2). Analysis of variance (ANOVA) when anti-Xa activity was grouped into three categories; sub-prophylactic (<0.2 U/ml), prophylactic (0.2-0.5 U/ml) and supra-prophylactic (>0.5 U/ml), demonstrated a difference of mean weights between the anti-Xa levels categories (Table 2). The sub-prophylactic group had the highest mean weights and the supra-prophylactic had the lowest mean weights (Figs. 3 and 4).

**Table 3:** Correlation of patient-related covariates and anti-Xa activity

Anti-Xa activity		
Age (years)	Pearson Correlation	0.127
	Sig. (2-tailed)	0.322
	N	63
BMI (kg/m^2^)	Pearson Correlation	-.422^a^
	Sig. (2-tailed)	0.001
	N	63
CR (mg/dl)	Pearson Correlation	-0.206
	Sig. (2-tailed)	0.105
	N	63
Weight (kg)	Pearson Correlation	-.602^a^
	Sig. (2-tailed)	0.0001
	N	63
CrCl (ml/min)	Pearson Correlation	-.355^a^
	Sig. (2-tailed)	0.004
	N	63
Height (m)	Pearson Correlation	-.353^a^
	Sig. (2-tailed)	0.005
	N	63

^a^Correlation is significant at the 0.01 level (2-tailed)

For Multivariate analysis we used Multivariate Linear Regression. The dependent variables were anti-Xa and the independent variables included in the regression were: female (as a dummy variable), BMI, CrCl and weight. Only weight and female sex were found to be significant (Table 4).

**Table 4:** Multivariate analysis of factors associated with anti-Xa activity

	B	Sig.	95.0% Confidence Interval for B	
			Lower Bound	Upper Bound
BMI (kg/m^2^)	-0.002	0.740	-0.012	0.0086
CrCl (ml/min)	0.000	0.384	-0.001	0.0005
Total body weight (kg)	-0.004	0.038	-0.007	-0.0002
Female	0.072	0.030	0.007	0.1359

R Square=0.443

## Discussion

The present study assessed a cohort of 63 rehabilitation patients recovering from either SAIS or SCI. Only 73% patients (46/63) receiving fixed-dose prophylactic enoxaparin 40mg achieved the recommended therapeutic target. More than one-quarter of patients (27%) in our study were found to be outside the recommended range for anti-Xa activity. Twelve patients (19%) had sub-prophylactic anti-Xa activity and five patients (7.9%) had supra-prophylactic levels of anti-Xa. Weight and CrCl were found to be the most influential factors on anti-Xa activity. The data showed a negative correlation between anti-Xa levels; as weight increased, the level of anti-Xa activity decreased to the sub-prophylactic range whereas lower CrCl correlated with supra-prophylactic anti-Xa activity for renal function. Being within the recommended therapeutic range for anti-Xa is especially important for these clinical subgroups, since patients recovering from SAIS or SCI are already at an increased risk of developing VTE or bleeding events compared to the general population.

The product information of enoxaparin states that there is an increase in exposure of enoxaparin with renal impairment and such patients should be monitored for signs of bleeding. In patients with renal impairment (CrCl <30ml/min), exposure to enoxaparin is significantly increased and dose adjustment is recommended [34]. In addition, the safety and efficacy of a fixed daily dose of 40 mg in patients with obesity (BMI>30) has not been determined and there is no consensus for dose adjustment. Similarly, dose adjustment is controversial for patients who are underweight. Underweight individuals may have increased exposure to enoxaparin and, therefore, a higher risk of bleeding while obese individuals are at a higher risk of thromboembolism [34]. Our study demonstrates that weight and CrCl do indeed correlate with anti-Xa activity. Nonetheless, this correlation can lead to non-therapeutic activity even in patients whose weight and creatine are within the recommended ranges.

Weight and CrCl demonstrated negative correlations. However, the multivariate analysis demonstrated multiple regressions with significant findings for weight and female sex. Weight is associated with volume of distribution and this was an expected result similar to other studies. Female was an unexpected finding however this may possibly be due to gender related differences in fat distribution and muscle mass.

Our study confirms previous reports showing that fixed-dose prophylactic regimens are often unsuitable for many patients [18–20, 23–29, 35, 36]. Similar to our results, Maurice-Dror et al recently reported that, among a cohort of 76 oncology patients, 16 (21%) demonstrated sub-prophylactic anti-Xa activity (<0.2 U/ml). These authors concluded that a substantial number of cancer patients receiving enoxaparin prophylaxis are undertreated, with weight and plasma pre-treatment coagulation parameters being influential factors [22]. Several other studies have also shown that different dosing strategies should be considered in patients who are underweight or overweight [21, 29, 37–39].

However, studies in different settings focusing on patients in trauma intensive care have reported higher percentages of patients with subtherapeutic anti-Xa activity [18, 35, 36, 40]. The ATLANTIC study, published by Rakhra et al, investigated anti-Xa levels in trauma intensive care patients hospitalized following traumatic brain injury (TBI) or SCI. In this study, 12/25 (48%) patients demonstrated low peak anti-Xa activity (≤0.2 U/ml) with fixed-dose prophylactic enoxaparin. The study concluded that high-risk critically ill patients receiving prophylactic enoxaparin often show inadequate anti-Xa activity and further investigation is required concerning dose optimisation [18].

There are several limitations to our study, including the small sample size. In addition, the data is only valid for the sub-groups tested and, while it may be logical to extrapolate to other groups, more research is required to ascertain the relevance of these findings. During the study period, there were no recorded episodes of major bleeding or VTE-related events. However, the study was not designed for a systematic evaluation of these events. Assessing VTE events can be complicated, as different assessments give different analyses. Moreover, VTE and bleeding events are relatively rare, and it would take a large number of patients to view statistically relevant results. Monitoring anti-Xa allows us to evaluate efficacy with a smaller cohort of patients.

## Conclusion

Our study demonstrates that a one-size-fits-all approach for VTE prophylaxis using a 40 mg/day regimen may be improper for rehabilitation patient populations. The efficacy of fixed-dose enoxaparin prophylaxis is limited and may be influenced by renal function and weight. Patients who are hospitalized for rehabilitation are especially sensitive to bleeding or thrombotic events that can further complicate their recovery. For this reason, anti-Xa studies should be considered in certain patients, such as those who underweight, overweight and have suboptimal renal function.

### Supplementary Information

**Additional file 1: Table S1.** Analysis of patient-related covariates and anti-Xa activity.

## Data Availability

The datasets used and/or analysed during the current study are available from the corresponding author on reasonable request.

## Abbreviations

ASH: American Society of Haematology
anti-Xa: Anti-factor Xa
AT: Antithrombin
BMI: Body mass index
CrCl: Creatinine clearance
DVT: Deep venous thrombosis
FXa: Factor Xa
LMWH: Low molecular weight heparin
PE: Pulmonary embolism
CR: Serum creatinine
SAIS: Sub-acute ischemic stroke
SCI: Spinal cord injury
UFH: Unfractionated Heparin
VTE: Venous thromboembolism
