# Supplementary material for: Enoxaparin for VTE thromboprophylaxis during inpatient rehabilitation care: assessment of the standard fixed dosing regimen
Source: BMC Pharmacol Toxicol. 2024 Jan 10;25:8. doi: 10.1186/s40360-023-00728-0 (PMC10782744; doi:10.1186/s40360-023-00728-0)
Supplement: Supplementary file 1 — Additional file 1: Table S1. Analysis of patient-related covariates and anti-Xa activity. [file 40360_2023_728_MOESM1_ESM.docx]

**Supplementary Material**

**S1: Analysis of patient-related covariates and anti-Xa activity**

|  | sex | N | Mean | Std. Deviation | p |
| --- | --- | --- | --- | --- | --- |
| Age (years) | Male | 44 | 57.14 | 15.200 | 0.048 |
|  | Female | 19 | 63.53 | 9.460 |  |
| Weight (kg) | Male | 44 | 79.648 | 13.6738 | 0.109 |
|  | Female | 19 | 72.995 | 17.4597 |  |
| BMI (kg/m^2^) | Male | 44 | 26.4495 | 4.22878 | 0.231 |
|  | Female | 19 | 28.0579 | 6.05423 |  |
| CR (mg/dl) | Male | 44 | 0.92045 | 0.171965 | 0.001 |
|  | Female | 19 | 0.76842 | 0.129326 |  |
| eGFR (ml/min/1.73m2) | Male | 44 | 102.4668 | 33.83668 | 0.174 |
|  | Female | 19 | 89.7500 | 33.31069 |  |
| ANTI_Xa (U/ml) | Male | 44 | 0.28318 | 0.108129 | 0.002 |
|  | Female | 19 | 0.38263 | 0.117985 |  |
| CrCl (ml/min/1.73m^2^) | Male | 44 | 102.10138 | 31.162753 | 0.012 |
|  | Female | 19 | 84.84992 | 20.469482 |  |
| Height (cm) | Male | 44 | 1.73531 | 0.081072 | 0.000 |
|  | Female | 19 | 1.61117 | 0.071862 |  |
